# IRAK4 Deficiency in a Patient with Recurrent Pneumococcal Infections: Case Report and Review of the Literature

**DOI:** 10.3389/fped.2017.00083

**Published:** 2017-04-28

**Authors:** Karina Gobin, Mary Hintermeyer, Bertrand Boisson, Maya Chrabieh, Pegah Ghandil, Anne Puel, Capucine Picard, Jean-Laurent Casanova, John Routes, James Verbsky

**Affiliations:** ^1^Division of Asthma, Allergy and Clinical Immunology, Department of Pediatrics, Medical College of Wisconsin, Milwaukee, WI, USA; ^2^Laboratory of Human Genetics of Infectious Diseases, Necker Branch, INSERM U1163, Imagine Institute, Paris, France; ^3^Paris Descartes University, Paris, France; ^4^St Giles Laboratory of Human Genetics of Infectious Diseases, Rockefeller Branch, New York, NY, USA; ^5^Department of Medical Genetics, School of Medicine, Ahvaz Jundishapur University of Medical Sciences, Ahvaz, Iran; ^6^Diabetes Research Center, Ahvaz Jundishapur University of Medical Sciences, Ahvaz, Iran; ^7^Pediatric Hematology-Immunology Unit, Assistance Publique Hôpitaux de Paris (AP-HP), Necker Hospital for Sick Children, Paris, France; ^8^Center for the Study of Primary Immunodeficiencies AP-HP, Necker Hospital for Sick Children, Paris, France; ^9^Howard Hughes Medical Institute,New York, NY, USA; ^10^Division of Rheumatology, Department of Pediatrics, Medical College of Wisconsin, Milwaukee, WI, USA

**Keywords:** IRAK4 deficiency, MYd88 deficiency, toll-like receptors, NF-κB essential modulator, NF-κB, IκBα

## Abstract

Primary immunodeficiencies are genetic defects of the innate or adaptive immune system, resulting in a propensity to infections. The innate immune system is the first line of defense against pathogens and is critical to recognize microbes and start the inflammatory cascade. Sensing of microbes occurs by a number of pathogen-recognition receptors, resulting in the activation of inflammatory signal transduction pathways, such as the activation of NF-κB. Herein, we describe a case of IRAK4 deficiency, a key signal transduction molecule of toll-like and IL-1 receptors. We highlight the complexities in diagnosis of these disorders and review genetic defects of the NF-κB pathway.

## Introduction

Primary immunodeficiencies consist of various genetic defects that affect the innate and adaptive immune systems, resulting in increased susceptibility to infections. The innate immune system is the first line of defense against a wide array of pathogens and initiates the inflammatory cascade that aids in the activation of the adaptive immune response (1). Various cells (e.g., neutrophils, macrophages, and NK cells), proteins (e.g., complement, cytokines), and receptors [e.g., toll-like receptors (TLRs) and other pattern-recognition receptors (PRRs)] comprise the innate immune system. The adaptive immune system, composed of T and B lymphocytes, develops after activation of the innate immune system and is critical to clear infections. Humoral or antibody-mediated immunity predominantly functions to eradicate extracellular infections, while cellular or T cell-mediated immunity is essential for intracellular pathogens (1). We report a case of defective innate immunity to highlight one presentation of these disorders, and the diagnostic challenge they can pose.

## Case Report

**Figure 1:**
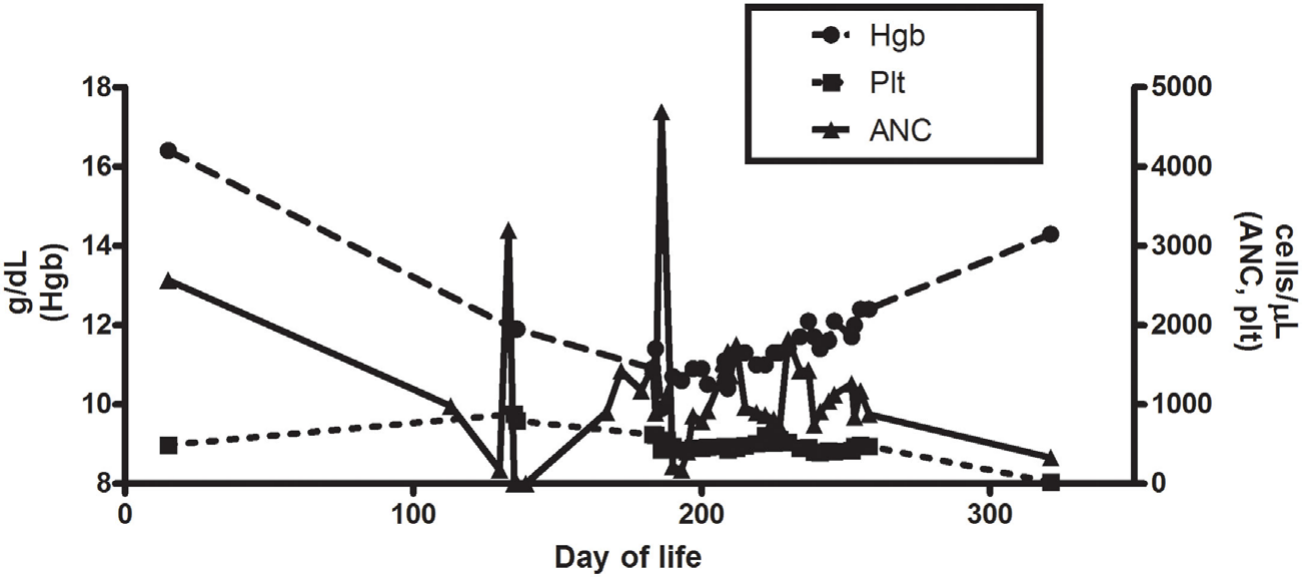
**Hemoglobin, platelet, and absolute neutrophil counts for patient during clinical course**.

A full-term, Caucasian, female infant was born to a healthy mother without complications. There was no family history of consanguinity, recurrent infections, or autoimmunity. She had a 5-year-old brother who was healthy. At 2 weeks of age, she presented to a hospital with fever to 38.3°C and irritability. A comprehensive evaluation for sepsis was initiated, and she was treated empirically with ampicillin and cefotaxime. Cerebrospinal fluid cultures were positive for enterovirus, and urine cultures demonstrated 10,000–100,000 colonies of *Escherichia coli*. A CRP at that time was normal. Voiding cystourethrogram and renal ultrasound, performed to evaluate possible secondary causes of the urinary tract infection, were normal. During admission, laboratory testing showed a white blood cell count (WBC) of 13.5 K/µL with an absolute neutrophil count (ANC) of 2,565 K/µL. Follow-up testing 3 days later showed a WBC count of 7.6 K/µL and ANC of 988 K/μL (Figure 1). One week after hospitalization, the infant had fully recovered.

The patient was well until 3 months of age when she developed left inguinal swelling. She was evaluated by her pediatrician with laboratory testing showing a WBC count of 8.9 K/µL and an ANC of 979 K/µL. The mass increased in size with erythema and induration noted, but patient had no fevers or other signs of systemic illness. Ultrasound demonstrated inflamed and possibly necrotic lymph node without evidence of abscess. The patient was admitted for administration of intravenous (IV) clindamycin, the swelling resolved, and she was discharged on oral clindamycin. Shortly after discharge, she had fevers and was diagnosed with otitis media, and her antibiotics were changed to amoxicillin–clavulanate. Despite treatment, she continued to have fevers up to 40°C, increasing erythema of the groin, and increased irritability. She was then hospitalized for IV antibiotics and incision and drainage, with cultures demonstrating *Staphylococcus aureus*. She responded appropriately to IV antibiotics and was sent home on oral amoxicillin–clavulanate.

The patient was seen for follow-up in her pediatrician’s office 3 days after discharge. She was afebrile, not irritable, and there was no sign of infection at her surgical site. However, blood culture from the previous admission was positive for *S. pneumoniae* after 5 days of growth. She received intramuscular ceftriaxone in the office and was directly admitted to the hospital for sepsis. A complete blood count (CBC) drawn at admission showed a WBC count of 9.3 K/µL with an ANC of 0 K/µL. To evaluate for neutropenia, neutrophil antibody testing was sent along with serial CBCs to rule out cyclic neutropenia. Abdominal and pelvic CTs were performed to exclude occult abscesses and only demonstrated reactive lymph nodes in the inguinal retroperitoneal area. With previously normal neutrophil counts with infection and absence of other cytopenias, it was thought that her current neutropenia was most likely related to consumption due to infection. The patient’s blood culture drawn at admission was negative, she was well appearing, and consequently discharged home on oral amoxicillin–clavulanate.

Three weeks later, the patient was diagnosed with bilateral otitis media treated with amoxicillin (WBC 6.4 K/µL with ANC of 900 K/µL). Two weeks later, she was diagnosed with right otitis media treated with cefdinir. WBC at this evaluation was 7.9 K/µL with an ANC of 1,422 K/µL. Although she was well appearing without fever, blood culture was drawn and was positive for *S. pneumoniae*, sensitive to cefdinir. Patient was admitted to the hospital and received IV ceftriaxone and was subsequently sent home on a course of oral cefdinir. Clindamycin prophylaxis was initiated against *S. pneumoniae* taking into account of the frequency and severity of the infant’s infections.

The patient had serial CBCs to evaluate for possible cyclic neutropenia. It was noted that her neutrophil counts were quite variable with nadirs ranging from 500 to 700 K/µL, but did not vary in a cyclical pattern (Figure 1). Anti-neutrophil antibodies were negative. DNA sequencing of the *ELA2* gene was normal. Serum immunoglobulins, pneumococcal antibody titers, lymphocyte subset analysis, expression of CD15 and CD18, and DHR were all normal (Table 1). Radiographs of the long bones, serum trypsinogen, and stool pancreatic elastase, sent to evaluate for Shwachman–Diamond syndrome, were normal (Table 1).

**Table 1:** **Immunologic and hematologic laboratory results**.

Pneumococcal vaccine titers	6/7 serotypes positive	
Neutrophil antibody panel	Negative	
Crossreactive autoneutrophil antibodies	Negative	
*ELA2* gene mutation	Negative	
IgG	446 mg/dL	174–857 mg/dL
IgA	23 mg/dL	10–75 mg/dL
IgM	37 mg/dL	22–95 mg/dL
IgE	17 mg/dL	24–85 mg/dL
ALC count	4,898/mm^3^	3,900–9,000/mm^3^
Absolute B cell count	1,567/mm^3^	430–3,000/mm^3^
Absolute T cell count	3,233/mm^3^	2,500–5,650/mm^3^
Absolute NK cells	147/mm^3^	170–830/mm^3^
T cell subset: CD3+CD4+	2,400/mm^3^	1,800–4,000/mm^3^
T cell subset: CD3+CD8+	686/mm^3^	590–1,600/mm^3^
T cell subset: CD4+CD45RA+	70%	
T cell subset: CD4+CD45RO+	8%	
B cell naïve/memory subset: CD27+	7%	
B cell naïve/memory subset: IgD+CD27−	89%	
B cell naïve/memory subset: IgD−CD27+	1%	
B cell naïve/memory subset: IgD+CD27+	6%	
B cell naïve/memory subset: CD21+CD27−	73%	
B cell naïve/memory subset: CD21−CD27+	2%	
B cell naïve/memory subset: CD21+CD27+	5%	

At the age of 6 months, she developed otitis media with accompanying *S. pneumoniae* bacteremia. Tympanostomy tubes were placed, and retrotympanic membrane fluid was positive for *S. pneumoniae*. She was treated with IV levofloxacin in the hospital, and then sent home on clindamycin prophylaxis. With the placement of tympanostomy tubes and clindamycin prophylaxis, the frequency of infections decreased, and her ANC trended upwards (Figure 1). With an improvement in her ANC and overall clinical picture, the patient was taken off of antibiotic prophylaxis.

At 11 months of age, patient presented to an ER with emesis and fever for 1 day. She was hospitalized for presumed gastroenteritis and received IV fluids. Shortly into the hospitalization, she became listless with poor perfusion and was transferred to the pediatric intensive care unit for respiratory failure. She was intubated and started on gentamicin and vancomycin and was found to have positive blood cultures for *S. pneumoniae*. She developed cardiogenic shock, multiorgan system dysfunction, and cerebral edema with poor perfusion. She was ultimately taken off life support and expired.

**Figure 2:**
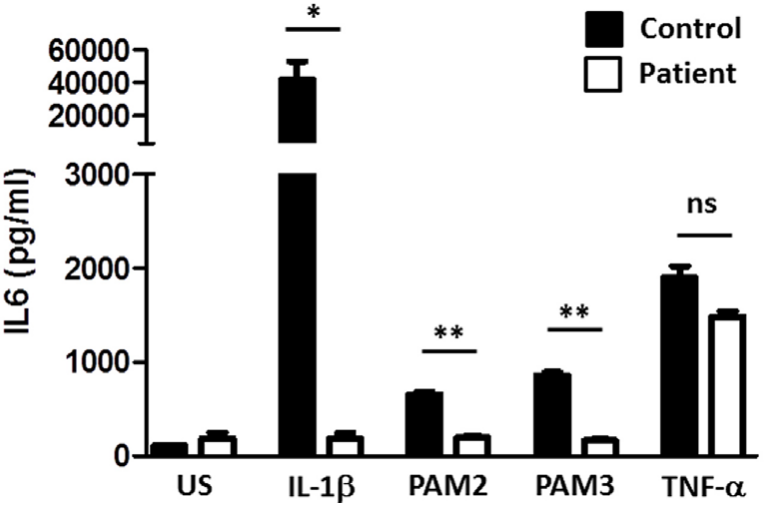
**Toll-like receptor (TLR) testing of patient fibroblasts**. SV40 transformed fibroblasts of the patient, and a healthy control were stimulated as indicated and IL6 production measured. PAM2 is a synthetic TLR2 agonist. PAM3 is a synthetic TLR1/2 agonist. A *t*-test was used to compare each agonist response of the patient versus a control (*<0.05, **<0.01).

Due to recent descriptions of genetic defects in the TLR pathway resulting in susceptibility to invasive infections with *S. pneumoniae* and *S. aureus*, postmortem genetic analysis was pursued demonstrating compound heterozygous mutation in the *IRAK4* gene (Q293X, 831+5G>T). IL1 stimulation of SV40 immortalized fibroblasts failed to induce normal amounts of IL6, whereas TNF-α stimulation was normal confirming loss of function of IRAK4 (Figure 2). Defective responses to other TLR agonists were also demonstrated.

## Discussion

This case highlights the presentation of TLR defects. TLRs are a widely expressed family of PRRs that respond to conserved microbial antigens, known as pathogen-associated molecular patterns, such as lipopolysaccharides (LPS), flagellin, and single-stranded RNA (2). Upon ligand binding, TLRs induce signaling transduction pathways leading to expression of gene products that initiate the inflammatory cascade. TLRs are single pass transmembrane proteins with extracellular leucine-rich repeat domains and a cytoplasmic tail that shows high similarity to the IL-1 receptor family. This portion is termed as the toll/IL-1 receptor (TIR) domain. In humans, 10 TLRs have been identified. TLR1, TLR2, TLR4, TLR5, TLR6, and TLR10 are extracellular, whereas TLR3, TLR7, TLR8, and TLR9 are located intracellularly within the endosome (1).

**Figure 3:**
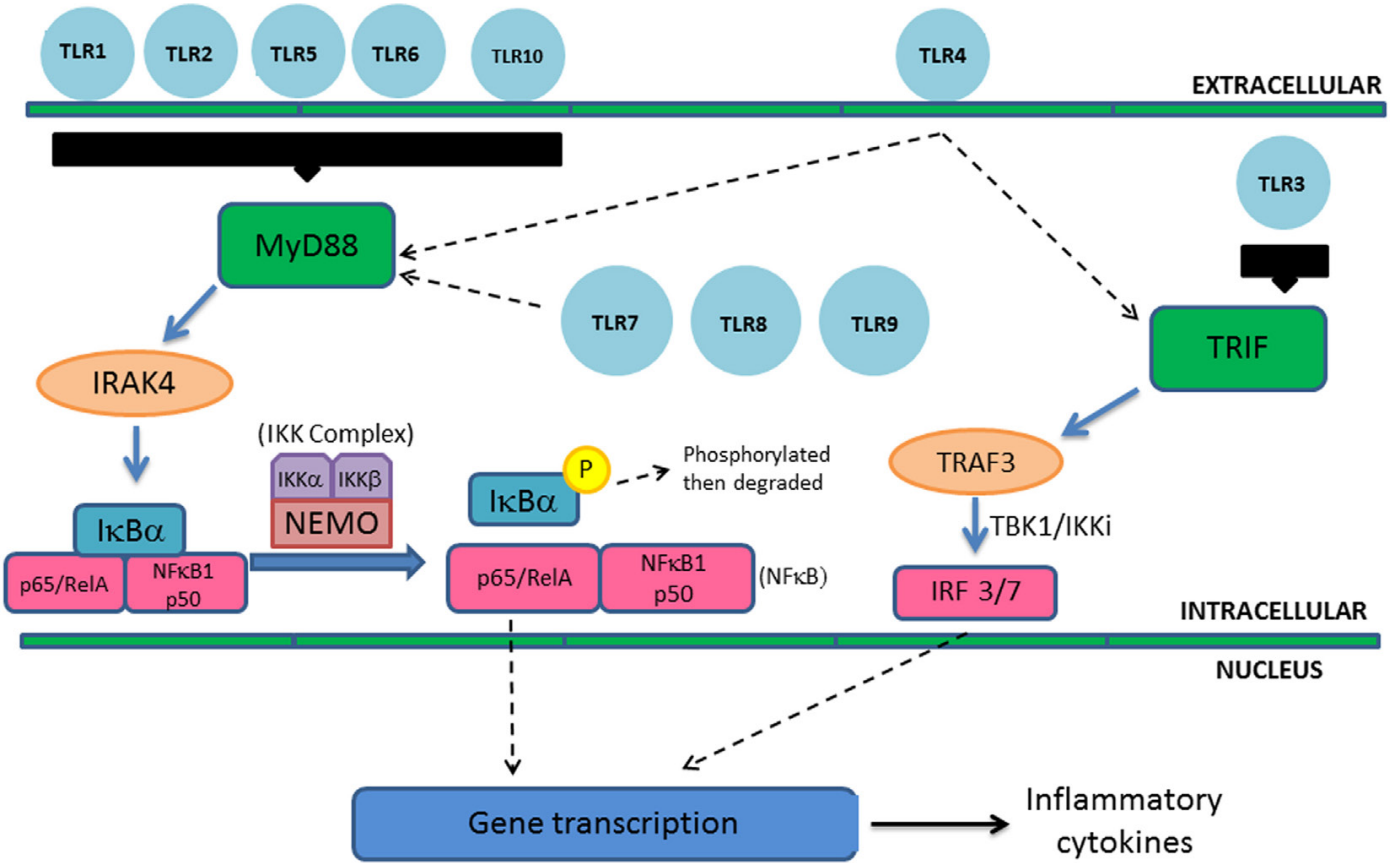
**Toll-like receptor signaling pathways**. Extracellular TLRs include TLR1, TLR2, TLR4, TLR5, TLR6, and TLR10 and TLR3, TLR7, TLR8, and TLR9 are located intracellularly within the endosome. Aside from TLR3, all other TLRs operate through MyD88 leading to activation of NF-κB and subsequent inflammatory cytokine gene transcription and expression. This is an example of canonical pathway utilization; however, other subunits can be activated. TLR3 operates through the TIR-domain-containing adaptor-inducing interferon-β pathway, while TLR4 can activate both pathways. MyD88 activates the IRAK complex (containing protein kinase IRAK4) leading to subsequent activation of the IκB kinase (IKK) complex. Activation of the IKK complex leads to phosphorylation and degradation of IκBα and translocation of NF-κB to the nucleus and production of inflammatory cytokines. TLR3 activation of TNF receptor-associated factor 3 results in transcription of type I interferons.

All TLRs except TLR3 utilize MyD88 as their cytosolic adaptor and trigger the canonical pathway leading to NF-κB activation and inflammatory cytokine gene transcription. MyD88 activates the IRAK complex, containing the protein kinase IRAK4, leading to activation of the IκB kinase (IKK) complex (Figure 3). The IKK complex includes NF-κB essential modulator (NEMO) and active IKKs (IKKα and IKKβ). NF-κB at baseline is bound to inhibitory proteins such as IκBα and IκBβ, sequestering it in the cytoplasm at baseline. Activated IKK complex leads to phosphorylation and degradation of IκBα and IκBβ, leading to translocation of NF-κB to the nucleus (3). Translocation leads to gene transcription and expression of various cytokines such as IL1β, IL-6, IL-12, IL-18, and TNF-α. TLR3 utilizes an alternative pathway *via* TIR-domain-containing adaptor-inducing interferon-β (TRIF) leading to the activation of TNF receptor-associated factor 3 (TRAF3) and expression of interferon regulatory factor 3 and 7. This leads to the transcription of type I interferons. TLR4 can trigger both the canonical and alternative pathways (4) (Figure 3).

### IRAK4/MyD88 Deficiency

Mutations in several genes involved in the TLR signaling pathways have demonstrated to cause primary immunodeficiencies in humans. IRAK4 and MyD88 are essential for signal transduction in the TLR canonical pathway. Consequently, IRAK4 and MyD88 deficiencies are indistinguishable clinically. IRAK4 and MyD88 deficiencies, which are inherited in an autosomal recessive manner, present with invasive severe pyogenic infections with *S. pneumoniae, S. aureus*, and *Pseudomonas aeruginosa* (5–8). Patients can present with meningitis, osteomyelitis, arthritis, abscesses, sepsis, and cellulitis. Affected individuals are not particularly susceptible to viral, parasitic, or fungal infections. Patients may mount a weak inflammatory response with delayed fever or minimal change in inflammatory markers (e.g., C reactive protein) with infections. Antibody levels, vaccines titers, and lymphocyte subpopulations are typically normal, although glycan-specific antibody response, including hemagglutinins and pneumococcal glycans, can be defective (9, 10). TLR testing with agonists such as LPS or IL-1β will show defective responses. As children age, the rate of infections usually decrease, which is likely due to the acquisition of humoral immunity and immunologic memory. There have been no deaths reported after 8 years of age in patients with IRAK4 deficiency (6), although there is a report of a 31-year-old with recurrent infections due to IRAK4 deficiency, suggesting that infectious episodes could continue throughout life (11). Despite being susceptible to a somewhat limited spectrum of bacteria, the mortality rate in these patients is 43%. Prophylactic antibiotic treatment, vaccinations against pyogenic bacteria, and immunoglobulin replacement starting early in life is recommended as prophylactic treatment (12).

### NEMO/IKK Complex Defects

Central to TLR signaling is the activation of NF-κB and defects in NF-κB signaling leads to immunodeficiencies. There are two pathways involved in NF-κB signaling: the canonical pathway that utilizes NEMO and the non-canonical pathway that uses NF-κB-inducing kinase. Activation of the canonical pathway results in the activation of the NEMO-dependent kinases, IKKα and IKKβ, which phosphorylate serine residues on IκBα and IκBβ resulting in their ubiquitination and degradation. Degradation of these inhibitors allows for NF-κB translocation into the nucleus and subsequent gene transcription (9, 13). Complete deficiency of NEMO is incompatible with life, while hypomorphic mutations result in an X-linked recessive disorder with immunodeficiency, anhidrosis, abnormal teeth, and ectodermal dysplasia (14, 15). The ectodermal defects in NEMO are due to the inability of the ectodysplasin A receptor to induce NF-κB activation. Patients with NEMO defects are susceptible to all pathogens, including invasive pyogenic infections with *S. pneumoniae, Haemophilus influenzae, S. aureus*, mycobacteria, fungi, and viruses. This disease is highly variable in its clinical presentation depending on the nature of the specific mutation. Patients have also been described to have osteopetrosis, vascular anomalies, autoimmunity, colitis, and arthritis. Routine labs in these patients can be normal; however, defective natural killer function and TLR signaling are the most consistent findings (16). Treatment includes antibiotic prophylaxis to prevent mycobacteria and *Pneumocystis*, and if patients have impaired B cell immunity, immunoglobulin therapy should also be administered. If there is functional B cell immunity, patients should be up to date on their vaccinations to *S. pneumoniae, H. influenzae*, and *Neisseria meningitidis* (12).

IκBα deficiency has a similar phenotype to NEMO, with ectodermal dysplasia and variable immunodeficiency, but exhibits autosomal dominant inheritance (17). Point mutations that affect the serine phosphorylation sites result in the most severe phenotype, with hypogammaglobulinemia, poor specific antibody production, and low proportions of CD4 and CD8 T cells. They are prone to recurrent bacterial infections including *S. aureus, S. pneumoniae, P. aeruginosa*, and *Salmonella enterica* and opportunistic infections like *Candidiasis* and *Pneumocystis jiroveci*. Autoimmunity can be seen in these patients as well, predominately manifested as recurrent diarrhea or colitis. Treatment also includes immunoglobulin therapy for impaired B cell immunity and antibiotic prophylaxis to *S. pneumoniae, H. influenza*, and *N. meningitidis* (12). Hematopoietic stem cell transplant has been completed with improvement of the primary immunodeficiency, but patients will persist with their ectodermal dysplasia (18). A milder form of this disease can be seen with certain nonsense mutations (19).

IκB kinase β, one of the kinases of the IKK complex responsible for phosphorylation of IκBα or IκBβ, results in an autosomal recessive immunodeficiency. Clinical presentation resembles both IκBα and NEMO mutations (3). Affected patients present in infancy with life-threatening bacterial, fungal, and viral infections and failure to thrive. Laboratory studies showed hypogammaglobulinemia with relatively normal numbers of circulating B and T cells, although the T cells in these patients are largely naive. T and B cell mitogen responses can be affected (20, 21).

The NF-κB complex is made up of homo- or heterodimers of several transcription factors: cRel, RelA/p65, RelB, p50, and p52. The p50 and p52 proteins are generated from longer precursors, p105 and p100, respectively. The p105 protein is encoded by *NFKB1*, whereas the p100 protein is encoded by *NFKB2*. Genetic defects in *NFKB1* or *NFKB2* result in common variable immunodeficiency with hypogammaglobulinemia, recurrent infections, and autoimmunity. Both of these disorders are inherited in an autosomal dominant manner, but exhibit variable penetrance and varying ages of presentation. *NFKB2* deficiency also results in central adrenal insufficiency and other pituitary defects (22–25).

In four patients of Cree ancestry from Canada with primary immunodeficiency-15 (IMD15; 615592), Pannicke et al. (20) identified a homozygous truncating mutation in the IKBKB gene (c.1292dupG; 603258.0001), resulting in complete loss of protein function. The mutation was found by homozygosity mapping followed by sequencing of genes in the candidate region. The patients presented in infancy with life-threatening bacterial, fungal, and viral infections and failure to thrive. Laboratory studies showed hypo- or agammaglobulinemia with relatively normal numbers of circulating B and T cells. Functional and gene expression studies of patient fibroblasts showed variable effects on receptor activation and NFKB signaling involved in immunity. There was impaired phosphorylation of NFKBIA (164008) in response to stimulation with TNFA (191160) and flagellin, which acts through TLR5 (603031), but only a marginally impaired response to IL1B (147720). IL6 (147620) response to TNFA was normal, but it was reduced in response to lipopolysaccharide, with acts through TLR4 (603030). These studies showed selective dependence of the regulation of NFKB target genes on IKBKB function. Patient peripheral blood B and T cells were almost exclusively of the naive type, and B, T, and NK cells showed poor differentiation or mitogenic responses under certain conditions. These findings were consistent with the role of IKBKB in transmitting signals by various surface receptors.

In a Turkish infant, born of consanguineous parents, with fatal IMD15, Nielsen et al. (21) identified a homozygous truncating mutation in the IKBKB gene (R272X; 603258.0002). The mutation was found by whole-exome sequencing. Western blot analysis of patient cells showed a complete lack of the IKBKB protein, although IKKA (CHUK; 600664) and NEMO (IKBKG; 300248) levels were similar to control. Stimulation of patient T cells failed to result in phosphorylation of p65 (NFKB3; 164014), and patient T cells failed to proliferate in response to stimulation. The findings indicated that IKBKB is critical for activation of T cells and differentiation of B cells.

### TLR3 Pathway Defects

Inborn errors of the TLR3 pathway can also result in immunodeficiency. TLR3-deficient patients suffer from recurrent herpes simplex encephalitis (26). TLR3 recognizes viral nucleic acids intracellularly, resulting in interferon production to limit virus spread (27). Mutations in TRAF3, TRIF, and UNC93B1 defects, proteins involved in signaling of TLR3, also result in recurrent herpes simplex encephalitis UNC93B1 is an intracellular endoplasmic reticulum protein involved in translocation of TLR3, TLR7, TLR8, and TLR9 into the endosome to meet with their ligands. This disorder is autosomal recessive and resulting in markedly lower levels of IFNα, IFNβ, and IFNγ in response to virus (28). Tumor necrosis factor TRAF3 is also involved in signaling through TLR3, and dominant-negative mutations in TLR3 result in impairment of binding to dsRNA and defective production of IFNα, IFNβ, and IFNγ (29). The low levels of IFNα, IFNβ, and IFNγ with viral stimulation result in high levels of viral replication and eventual cell death (26). TRIF specifically interacts with TLR3 and is required for signal transduction of this pathway. TRIF is also involved in TLR4 signaling. TRIF deficiency is autosomal recessive or autosomal dominant, presents with herpes encephalitis, and fibroblast from these patients demonstrated defective interferon and IL-6 production in response to TLR3 agonists (30). Function of TLR3, UNC93B1, TRIF, and TRAF3 appears to be crucial in neurons to control virus, but is expendable in other tissues. Diagnostic testing can be difficult as neuronal cells or fibroblasts will show a defect when tested, but peripheral blood mononuclear cell responses can be normal. Treatment with interferon along with acyclovir may improve prognosis (29).

Our patient had recurrent *S. pneumoniae* infections despite a largely normal immune workup, and without significant signs of infections, such as fever, even during episodes of bacteremia. This case highlights the importance of evaluating TLR signaling in any infant with recurrent or severe pyogenic infections. Recognition of these disorders is crucial as these patients present early in infancy often with blunted inflammatory responses that can lead to delayed diagnosis and grave outcomes.

## Ethics Statement

This study was carried out in accordance with the recommendations of the Children’s Hospital of Wisconsin Institutional Review Board with written informed consent from all subjects. As a single case report, this work was exempt from IRB review.

## Author Contributions

KG was the primary author and gathered all of the information and wrote the manuscript. MH was involved in obtaining and analyzing clinical data. JR and JV aided with manuscript preparation and review. BB, MC, PG, AP, CP, and J-LC did all of the genetic testing and functional testing reported in the manuscript.

## Conflict of Interest Statement

The authors declare that the research was conducted in the absence of any commercial or financial relationships that could be construed as a potential conflict of interest.
