# Corrigendum: IRAK4 Deficiency in a Patient with Recurrent Pneumococcal Infections: Case Report and Review of the Literature

**DOI:** 10.3389/fped.2018.00042

**Published:** 2018-03-02

**Authors:** Karina S. Gobin, Mary Hintermeyer, Bertrand Boisson, Maya Chrabieh, Pegah Ghandil, Anne Puel, Capucine Picard, Jean-Laurent Casanova, John Routes, James Verbsky

**Affiliations:** ^1^Division of Asthma, Allergy and Clinical Immunology, Department of Pediatrics, Medical College of Wisconsin, Milwaukee, WI, United States; ^2^Laboratory of Human Genetics of Infectious Diseases, Necker Branch, INSERM U1163, Imagine Institute, Paris, France; ^3^Paris Descartes University, Paris, France; ^4^St. Giles Laboratory of Human Genetics of Infectious Diseases, Rockefeller Branch, New York, NY, United States; ^5^Department of Medical Genetics, School of Medicine, Ahvaz Jundishapur University of Medical Sciences, Ahvaz, Iran; ^6^Diabetes Research Center, Ahvaz Jundishapur University of Medical Sciences, Ahvaz, Iran; ^7^Pediatric Hematology-Immunology Unit, Assistance Publique Hôpitaux de Paris (AP-HP), Necker Hospital for Sick Children, Paris, France; ^8^Center for the Study of Primary Immunodeficiencies AP-HP, Necker Hospital for Sick Children, Paris, France; ^9^Howard Hughes Medical Institute, New York, NY, United States; ^10^Division of Rheumatology, Department of Pediatrics, Medical College of Wisconsin, Milwaukee, WI, United States

**Keywords:** IRAK4 deficiency, MYd88 deficiency, toll-like receptors, NF-κB essential modulator, NF-κB, IκBα

In the published article, an author surname was incorrectly spelled as Gandil. The correct spelling is Ghandil. Anne Puel’s given name was incorrectly spelled as Ann. The correct spelling is Anne.

There was an error regarding the affiliations for Pegah Ghandil. As well as having affiliations 2 and 3, Pegah Ghandil should also have two more affiliations “Department of Medical Genetics, School of Medicine, Ahvaz Jundishapur University of Medical Sciences, Ahvaz, Iran” and “Diabetes Research Center, Ahvaz Jundishapur University of Medical Sciences, Ahvaz, Iran”.

The authors apologize for these errors and state that this does not change the scientific conclusions of the article in any way.

The original article has been updated.

## Conflict of Interest Statement

The authors declare that the research was conducted in the absence of any commercial or financial relationships that could be construed as a potential conflict of interest.

